# Immunoinformatics Predictions on Variable *Mycobacterium tuberculosis* Lineage 6 T Cell Epitopes and HLA Interactions in West Africa

**DOI:** 10.3390/microorganisms13051032

**Published:** 2025-04-29

**Authors:** Marta L. Silva, Nuno S. Osório, Margarida Saraiva

**Affiliations:** 1 i3S—Instituto de Investigação e Inovação em Saúde, 4200-135 Porto, Portugal; marta.silva@i3s.up.pt; 2 Doctoral Program in Molecular and Cellular Biology, Instituto de Ciências Biomédicas Abel Salazar (ICBAS), Universidade do Porto, 4050-313 Porto, Portugal; 3 Life and Health Sciences Research Institute (ICVS), School of Medicine, Campus Gualtar, University of Minho, 4710-057 Braga, Portugal; 4 ICVS/3B’s—PT Government Associate Laboratory, 4710-057 Braga, Portugal; 5 IBMC—Instituto de Biologia Molecular e Celular, Universidade do Porto, 4200-135 Porto, Portugal

**Keywords:** *Mycobacterium tuberculosis*, phylogeographic lineages, antigens, HLA binding affinity, host–pathogen interactions, co-evolution

## Abstract

Tuberculosis (TB), caused by *Mycobacterium tuberculosis* (Mtb), remains a global health challenge. The human-adapted TB-causing bacteria are distributed into ten lineages with distinct global distributions and clinical outcomes. Mtb lineages 4 (L4) and L6 are good prototypes of these differences, because L4 is globally prevalent, whereas L6 is geographically restricted to West Africa and associated with slower disease progression. Given the fundamental role of T cells for the control of TB, we questioned whether Mtb L4 or L6 antigens and HLA interactions would be disrupted in West African hosts. Here, we selected variable and validated antigens and demonstrate their expression during in vivo Mtb L4 or L6 infections. We then compared the predicted number of IFN-γ-inducing and HLA high-binding-affinity peptides in Mtb ancestral, L4, or L6 proteins, considering HLA alleles of high or low frequency in West Africa. Our immunoinformatics approach predicts that non-synonymous substitutions of high variance in Mtb L6 strains diminish binding affinities to HLA alleles prevalent in West African populations, suggesting specific adaptations of these strains to their preferred hosts. Future functional studies will advance our knowledge on lineage-specific evolution and inform strategies to enhance TB control in endemic regions.

## 1. Introduction

Tuberculosis (TB) remains a health problem, with an estimated 10.8 million new cases and 1.25 million deaths reported in 2023 alone [1] The causative agent of TB, Mycobacterium tuberculosis (Mtb), is an obligate human pathogen that likely originated thousands of years ago [2,3,4] and that belongs to the Mycobacterium tuberculosis complex (MTBC) [5]. Selective pressures from host immune responses and environmental factors have driven the diversification of Mtb into ten phylogenetic lineages (L1–L10) [5,6] that show a striking geographic structuring, even in the face of increased human mobility and globalization [3,5]. For example, MTBC L4 is globally distributed [7], displaying high incidence across Europe, Africa, and the Americas, while L2 dominates in East Asia [8]. In contrast, MTBC L5, L6, and L9 (formerly known as *Mycobacterium africanum* [9]) are largely restricted to specific regions of West and East Africa [10,11,12]. In these regions, MTBC L5, L6, and L9 may co-exist with globally spread lineages, most notably L4, without evidence of being out-competed [11,12].

Host–Mtb sympatry highlights the adaptation of the pathogen to specific hosts, and may be explained by different mechanisms, including co-evolution [13], genetic drift [14], and host restriction [15,16,17]. Several studies reported specific association of Mtb L5 infections with the Ewe ethnic group [18,19,20], with an effect visible on macrophage responses [21,22]. Furthermore, Mtb strains of L6 displayed reduced infectivity in allopatric macrophages and reduced transmission in allopatric populations [15] despite similar transmission rates in its West African niche [23]. A major gap in knowledge relates to the impact of Mtb–host sympatric adaptations in T cell responses, which are key players for TB control in humans and experimental models [24,25]. Mtb T cell epitopes are generally hyperconserved, a feature that may however be disrupted in Mtb L6, as T cell epitopes in Mtb strains of this lineage are more variable, as shown by a significantly higher average nucleotide diversity and a greater number of single nucleotide polymorphisms (SNPs) between sequences [10,26,27]. Furthermore, T cells from TB patients infected with Mtb L6 strains showed reduced production of key cytokines, such as interferon (IFN)-γ and interleukin (IL)-2, in response to antigens like ESAT-6 and CFP-10 [28], which are critical for robust T cell-mediated immunity [29,30,31]. This reduced immune activation may potentially explain the ability of Mtb L6 to cause TB disease despite being a slow-growing pathogen [32,33]. On the other hand, it could also lead to decreased inflammation and less tissue damage, allowing the pathogen to persist in the host while minimizing detection.

We hypothesized that the higher antigen variability in Mtb L6 may reflect an evolutionary adaptation of this lineage to evade immune recognition by human leukocyte antigen (HLA) molecules predominant in West African populations. This hypothesis would help to explain the geographic restriction of Mtb L6, whilst also fitting with the high sympatry of Mtb L6 pathogens and their West African host niche [11,12]. Leveraging the potential of immunoinformatics as a transformative tool in immunology [34,35,36], we focused on a set of seven experimentally validated antigens [27], previously described as variable in Mtb L6 genomic sequences, and conducted in silico analyses of HLA binding affinity and predictions of functional immunogenicity. The Mtb L6 variant proteins were predicted to associate with a reduction in the number of high-binding-affinity peptides to both HLA class I and class II alleles of high frequency in West African populations. In contrast, no predicted trend for increased or reduced binding affinity was detected for the Mtb L4 version of the candidate proteins, irrespective of the tested HLA. These in silico findings warrant future experimental validation so that they may potentially impact the development of more efficient TB vaccines, informed by host–pathogen interactions.

## 2. Materials and Methods

### 2.1. Ethics Statement

Animal experiments complied with the ARRIVE guidelines, followed the 2010/63/EU Directive and were approved by the i3S Animal Ethics Committee and the Portuguese National Authority for Animal Health (DGAV; #018413/2021-11-24).

### 2.2. Animal Housing and Infection

C57BL/6 mice were bred and housed at the i3s animal facility. Males and females of 8 to 11 weeks old were used for infections, and maintained under contention conditions in the animal biosafety level 3 facility at i3s, at a controlled temperature (20–24 °C) and humidity (45–65%), and a light cycle of 12 h (light/dark). Water and food were provided ad libitum. Mice were infected with Mtb (clinical isolates from L4 or L6) through the aerosol route using an inhalation exposure system (Glas-Col) [37,38]. To determine the dose of infection, the bacterial load in the lungs of 3 mice was quantified 3 days post-infection. Infected mice were weighed every week. Mice were euthanized by CO_2_ inhalation at the indicated time points.

### 2.3. Lung Processing, CFU Determination, and mRNA Analysis

Lungs were aseptically excised and processed as previously described [37,38]. Briefly, lungs were digested with Collagenase D (Roche, Basel, Switzerland, Cat. #11088882001) followed by physical disruption and filtration through 70 μM cell strainers (Falcon, Corning, NY, USA Cat. #352350.0). Cell suspensions were used for colony forming unit (CFU) determination and mRNA analysis. Lung cell suspensions were lysed with saponin serial dilutions prepared and plated in Middlebrook 7H11 agar (BD Biosciences, Franklin Lakes, NJ, USA, Cat. #212203.0) supplemented with OADC and PANTA (BD BioSciences, Franklin Lakes, NJ, USA, Cat. #245114). CFUs were enumerated after 21–28 days of incubation at 37 °C. Total RNA was extracted from infected mouse lungs using TRIzol reagent (GRiSP, Porto, Portugal, Cat. #GB23.0100) according to the manufacturer’s instructions. Briefly, samples were stored in TRIzol at −80 °C, homogenized, and subjected to phase separation using chloroform. RNA was precipitated with isopropanol, washed in 70% ethanol, and resuspended in RNase/DNase-free water. Glycogen was used as a carrier to enhance RNA yield. RNA concentration and purity were assessed by Nanodrop spectrophotometry. For qPCR analysis, cDNA was synthesized with the SuperScript First-Strand Synthesis System using Random Primer Mix (60 µM) for RT-PCR (ThermoScientific, Waltham, MA, USA, Cat. #E6300L). Target gene mRNA expression was quantified by real-time PCR, using SYBR green (Biorad, Hercules, CA, USA, Cat. #1725121). Gene expression was normalized to SigA. Oligonucleotide sequences are listed in Supplementary Table S1. Data from the qPCR (Figure 2) were analyzed using GraphPad Prism software v10.1.1. Differences were considered significant at *p* ≤ 0.05 and represented as follows: * *p* ≤ 0.05; ** *p* ≤ 0.01; and *** *p* ≤ 0.001.

### 2.4. Dataset and Genome Sequence Analysis

Publicly available MTBC genome sequences from Napier et al. were utilized [39]. A subset of 247 genomes, comprehensively representing Mtb L6 and diverse MTBC lineages, was selected. Raw sequencing reads were downloaded from the NCBI SRA and processed using a standardized bioinformatic pipeline. This pipeline included quality control with BBDuk (version 39.01); mapping to the inferred Mtb ancestral reference genome sequence (MTB_anc.fasta) derived from Comas et al. [2] using BWA-MEM (version 0.7.17); duplicate marking with samtools markdup (version 1.17); and targeted variant calling focused on specific regions of interest using bcftools mpileup (parameters --min-MQ 30, --min-BQ 20) and bcftools call (version 1.17). Variant filtering was applied using bcftools filter (version 1.17) to remove variants flagged as “LowQual” (criteria: QUAL < 15). Functional annotation of variants was performed using bcftools csq (version 1.17) with the GFF3 annotation file corresponding to the Mtb H37Rv reference genome (NC_000962.3).

### 2.5. Variant Allele Frequency Analysis and Visualization

Variants identified within the target genes of interest were extracted from the annotated VCF files. For each variant position in each sample, the variant allele frequency (VAF) was calculated using the DP4 (depth per allele) field from the VCF output of bcftools mpileup. This VAF represents the proportion of reads supporting the alternative allele at a given variant position. VAF values were analyzed using Python (version 3.12.2) with the pandas (version 2.2.3) and numpy (version 2.2.4) libraries. To identify variants differing significantly in frequency between lineages, the mean VAF per genomic position was calculated separately for Mtb L6 and L4 isolates, utilizing the lineage classifications provided in [40]. The difference between the mean VAF in Mtb L6 and the mean VAF in Mtb L4 was computed for each variant position. Variants were then ranked based on this VAF difference (VAF_Difference_L6_L4) to identify positions with the largest frequency discrepancies. Distributions of VAF values for selected high-difference variants across specific lineages were visualized using heatmaps generated with the seaborn (version 0.13.2) and matplotlib (version 3.10.1) libraries in Python. These heatmaps displayed the mean VAF per variant position (columns) for each lineage (rows).

### 2.6. IFNepitope Analysis

The IFNepitope server (https://webs.iiitd.edu.in, IFNepitope, module “Scan”, accessed on 23 March 2025) [40] was utilized to predict IFN-γ inducing epitopes for Mtb L6 and L4 candidate gene variants and the corresponding ancestral sequences, considering a length of 15 residues. This tool enables the identification of IFN-γ-inducing epitopes from a set of peptide sequences or a peptide library [41]. The search model employed was the support vector machine (SVM) motif hybrid, which integrates the SVM-based model with motif contributions relevant to IFN-γ induction.

### 2.7. HLA Selection for T Cell Epitope Prediction

Allele frequency data were retrieved from the publicly available database allelefrequencies.net to identify HLA alleles of high or low frequency in West Africa, as compared to other regions (Europe, North America, and Asia). In parallel, data from the Immune Epitope Database (IEDB; www.iedb.org) were consulted to confirm the availability of experimental binding data and to aid in the verification of allele representation in the target populations.

### 2.8. T Cell Epitope Prediction

Peptides of different lengths (8 to 14 mers for HLA class I and 9 to 18 mers for HLA class II) were generated for each candidate antigen. NetMHCpan-4.1b and NetMHCIIpan-4.0 were used to predict the binding potential of ancestral, Mtb L4, and Mtb L6 peptide variants to the selected HLAs. These machine learning-based tools were trained with a comprehensive number of mass spectrometry eluted ligands and have been considered the best overall predictors [42,43,44], namely in the HLA-DR locus [44]. HLA-binders were selected based on the half maximal inhibitory concentrations (IC_50_). IC_50_ < 50 nM was used as the cutoff for high-binding-affinity peptides.

## 3. Results and Discussion

### 3.1. Selection of Highly Variable Antigens in Mtb L6 Strains

**Figure 1:**
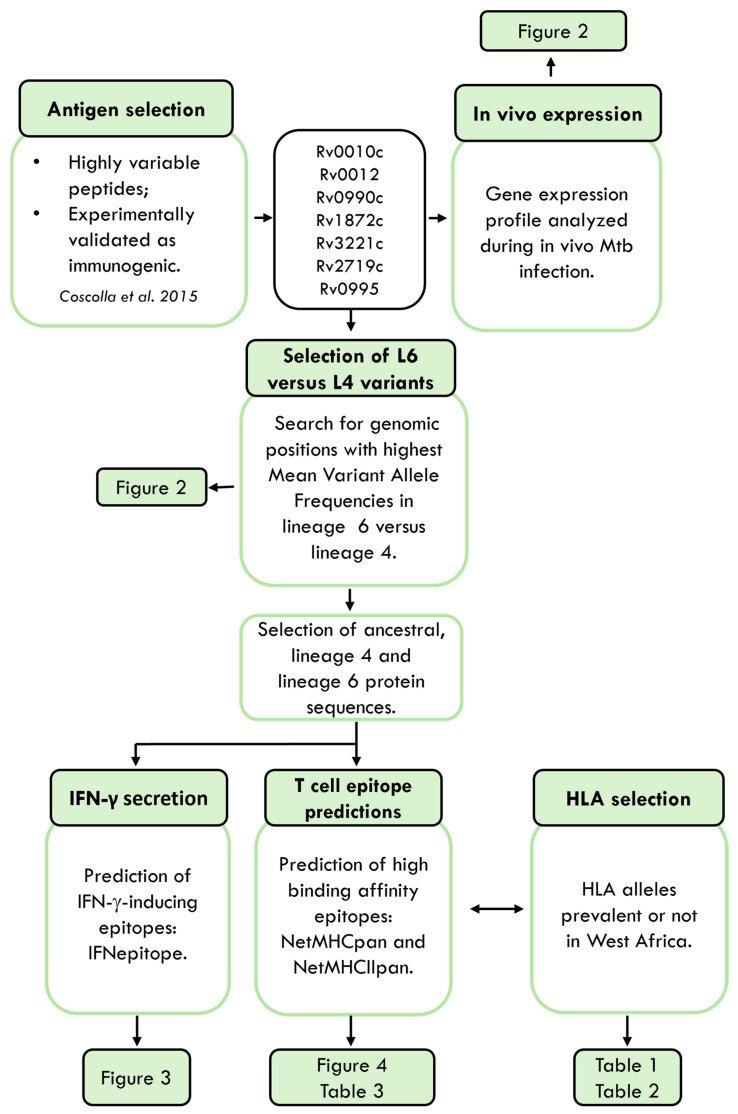
Schematic representation of the study. Mtb, *Mycobacterium tuberculosis*. HLA, human leucocyte antigen. Antigens were retrieved from [27].

Given the sympatry between Mtb isolates belonging to L6 and their West African hosts [11,12,15], we hypothesized that specific host–pathogen interactions may be in place and mediated by alterations in epitope recognition by HLAs. To investigate this hypothesis, we followed the approach summarized in Figure 1.

**Figure 2:**
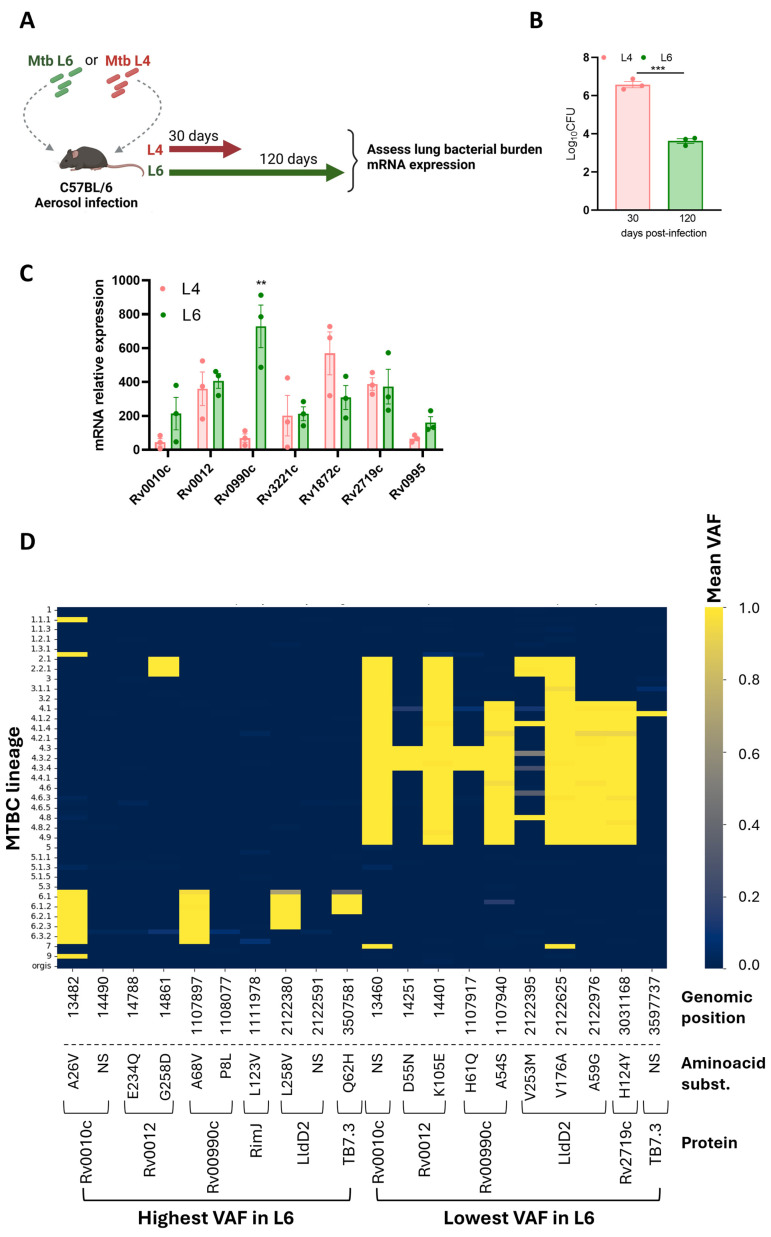
Expression and selection of variant antigens. (**A**) Experimental infection model. C57BL/6 mice were infected via aerosol with Mtb lineage 4 or lineage 6, and at the indicated time points, lung bacterial burdens (**B**) and candidate gene expression (**C**) were assessed. Data are represented as mean ± SEM. Each dot represents an individual mouse. Statistical analysis was performed using 2-tailed unpaired Student’s *t* test. (**D**) Heat map representing the genomic regions with the highest and lowest mean variant allele frequencies (VAFs) in Mtb L6 compared to Mtb L4. L, lineage. Mtb, *Mycobacterium tuberculosis*. MTBC, *Mycobacterium tuberculosis* complex. Ns represents synonymous substitutions. Differences were considered significant at *p* ≤ 0.05 and represented as follows: ** *p* ≤ 0.01; and *** *p* ≤ 0.001.

We reasoned that particular epitope–HLA interactions would be more likely for the rare variable Mtb antigens than for the more common hyperconserved ones. Thus, we selected for our study seven proteins previously identified as rare variant epitopes across 216 Mtb strains and experimentally validated as immunogenic in samples from individuals with active TB [27]. Although epitopes for the selected proteins had been tested for their immunogenicity in human samples [27], whether the corresponding genes were expressed during Mtb L6 infections had not been tested. We aerosol-infected C57BL/6 mice with a Mtb clinical isolate belonging to L6 [33] or with one belonging to L4 [45]. Analyses were performed at day 120 or day 30 post-infection for the Mtb L6 or Mtb L4 infection, respectively (Figure 2A). At these time points, the infections reach a chronic phase with stabilized bacterial burdens. As expected from previous studies [33], the bacterial burden in the slow-progressing Mtb L6-infected mice was lower than that observed in Mtb L4-infected mice (Figure 2B). The transcriptional analysis of the candidate genes by qRT-PCR showed their in vivo expression, irrespective of the Mtb isolate used (Figure 2C). The pattern of expression was similar for all tested genes, with the exception of Rv0990c, which at the indicated time points displayed higher expression in the lungs of mice infected with the Mtb L6 isolate as compared to Mtb L4 infections (Figure 2C). While B6 mice may not be the best models to study TB pathogenesis, our findings show that the selected genes are expressed in the lungs of Mtb-infected mice in the presence of a protective immune response, which positions them as possible natural antigens.

The genes encoding these seven proteins were inspected for genomic variants using a collection of 247 MTBC genomes from [39] that comprehensively represent L6 and diverse MTBC lineages. For each sample and genomic position within these genes, we determined the presence of variants and calculated their VAF. Mean VAF analysis across all lineages revealed a predominantly low VAF across the majority of positions (Supplementary Table S2), consistent with the expected conservation of T cell epitopes in Mtb [26,27]. To specifically highlight variants with differential prevalence between L6 and L4, we ranked genomic positions based on the difference in mean VAF between these two lineages. To visualize the top 10 L6 versus L4 VAF patterns, we generated a heatmap (Figure 2D) displaying mean VAF for each genomic position (columns) across different MTBC lineage level 3 classifications (rows). The color intensity in each cell corresponds to the mean VAF for that position within that lineage group, ranging from dark blue for low VAF to bright yellow for high VAF (Figure 2D). Four genomic mutations identified as high mean VAF in L6 versus L4 strains, or vice versa, led to non-synonymous amino acid substitutions in the corresponding proteins (Figure 2D). Genomic positions 1107897 (containing the SNP resulting in the A68V substitution in protein Rv0990c), 2122380 (containing the SNP resulting in the L258V substitution in protein LldD2/Rv1872c), and 3597581 (containing the SNP resulting in the Q62H substitution in protein Rv3221c) exhibited markedly elevated mean VAF values in several L6 sub-lineages (e.g., 6, 6.1, 6.2, 6.3) compared to other lineages shown in the heatmap (Figure 2D). On the other hand, the non-synonymous substitutions in Rv0010c, Rv0012, RimJ, and P8L in Rv00990c exhibited elevated mean VAF values in only one of the L6 sub-lineages (Figure 2D). Thus, while the selected proteins are generally conserved across MTBC, specific genomic positions within these candidate genes display increased variability specifically within L6 strains. These positions, characterized by higher VAF in L6, may represent sites under positive selection within this lineage, potentially influencing epitope presentation and subsequent T cell recognition within West African populations. Regarding the mutations detected as high mean VAF in L4 versus L6, we also identified some that are L4-specific and others that also span other lineages, most commonly L2 and L3 (Figure 2D).

### 3.2. Immunogenicity Predictions for the Variant Epitopes

**Figure 3:**
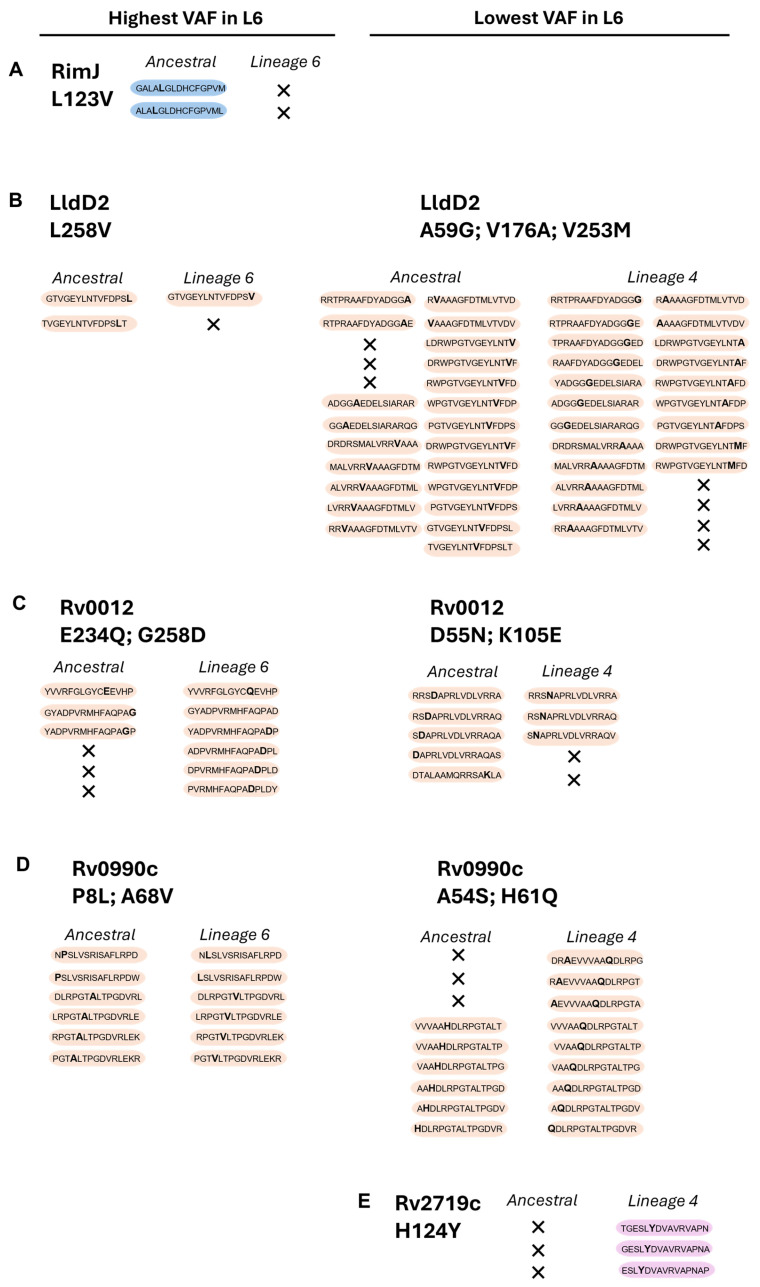
Prediction of IFN-y-inducing epitopes for the selected proteins. Data represent computational predictions performed using IFNepitope (SVM) to discriminate IFN-y-inducing epitopes. These are selected based on reaching a predicted positive score. Non-synonymous substitutions and predicted IFN-y-inducing epitopes are indicated for the selected proteins: RimJ (**A**), LldD2 (**B**), Rv0012 (**C**), Rv0990c (**D**), and Rv2719c (**E**), across the two Mtb variants (L4 and L6) in comparison with the ancestral sequence. In each peptide, the non-synonymous amino acid substitution is represented in bold; X represents loss of IFN-γ-inducing epitopes. Peptides with non-synonymous substitutions only in Mtb L6 are shaded blue; those common to both Mtb L6 and L4 are shaded orange; and those only in Mtb L4 are shaded pink.

We then assessed the predicted potential of the ancestral, Mtb L4, and Mtb L6 variant proteins to elicit IFN-γ responses. We used the IFNepitope tool to predict IFN-γ-inducing peptides (15 amino acid residues) across the 10 highest or lowest VAFs in Mtb L6 strains, causing non-synonymous substitutions (Figure 2D). Of the top 10 L6 VAFs, Rv0010c and TB7.3 displayed no predicted IFN-γ-inducing epitopes that included the mutated sites. The pattern for the other candidate L6 variants varied, with a loss of predicted IFN-γ-inducing epitopes in RimJ (Figure 3A) and LldD2 (Figure 3B), a gain of epitopes in the case of Rv0012 (Figure 3C), and no change for Rv0990c (Figure 3D). In the case of the variants with a low VAF in Mtb L6, a mixed pattern of increase and decrease was detected for LldD2, depending on the amino acid substitution (Figure 3B), while Rv0012 showed a predicted decrease (Figure 3C), and an increase in the number of predicted IFN-γ-inducing peptides was detected for the Mtb L4 variants of Rv0990c (Figure 3D) and Rv2719c (Figure 3E). Therefore, no specific pattern of gain or loss of predicted IFN-γ-inducing epitopes was detected as being common across each protein or for each lineage. This suggests that any potential functional impact of the antigen variants may be primarily dependent on the binding of the variable peptide to the host HLA. Importantly, HLA genes are highly polymorphic in human populations and several alleles have been previously associated with TB susceptibility in different populations [46,47,48,49,50,51,52,53,54]. Thus, it is possible that the binding affinity of the variable antigens to the matching host HLAs may vary, resulting in altered T cell responses.

### 3.3. HLA Binding Affinity Predictions for Mtb L6 Epitopes

To investigate whether the peptide variants of highest or lowest prevalence in Mtb L6 versus L4 would impact their predicted interactions with host HLAs, we started by retrieving a comprehensive set of HLA alleles, based on the geographic restriction of Mtb L6 to West Africa [10,11,12] and the global prevalence of L4 [7]. From the 10387 HLA class I alleles available in NetMHCpan-4.1, and the 660 HLA-DRB alleles (class II) available in NetMHCIIpan-4.0, we selected those with

1. high frequency in at least one of the five West African countries (Guinea-Bissau, Mali, Senegal, The Gambia, and Burkina Faso), but not at a global level;
2. high frequency in Europe, North America, and Asia, and low frequency in West African countries.

We excluded HLA-C class I alleles due to their limited polymorphism and lower expression levels (about 90% less) compared to the other two loci [55,56,57]. Within the HLA class II -DR locus, only the DRB1 alleles were selected, as they bind most Mtb epitopes and were previously predicted as displaying a five-times-higher expression in comparison to other alleles [58]. HLA-DP and HLA-DQ class II loci were not considered for the study due to the lack of experimental binding data available [59]. Thus, we selected five HLA-A and five HLA-B class I alleles (Table 1), and six HLA-DRB1 class II alleles (Table 2) of high or low frequency in West Africa.

Using computational predictions from NetMHCpan-4.1 and NetMHCIIpan-4.0, we assessed the number of high-binding-affinity epitopes (IC_50_ < 50 nM) of the ancestral or variant protein for each selected HLA. Different patterns of variation were detected between the Mtb L6 or L4 variants and the ancestral haplotype, depending on the HLA/peptide pair. Non-synonymous variants of Rv0010c, which were only detected for Mtb L6, did not impact the number of high-binding-affinity epitopes predicted for class I HLAs but reduced the predicted number of high-binding-affinity epitopes for HLA class II alleles, independently of them having high or low prevalence in West Africa (Table 3). In the case of RimJ, the identified non-synonymous variant of L6 did not impact the predicted number of high-binding-affinity epitopes to either class of HLA (Table 3). The last Mtb L6 unique non-synonymous mutation, in TB7.3, led to a loss in the predicted number of high-binding-affinity epitopes only to HLA class I and only for those highly prevalent in West Africa (Table 3). Regarding Rv0012, Rv0990c, and LldD2, the three proteins showing non-synonymous mutations in both Mtb L6 and Mtb L4 strains, a pattern of loss of the predicted number of high-binding-affinity epitopes for the Mtb L6 variants to HLA class I and II alleles of high prevalence in West Africa was detected (Table 3). However, this pattern was not recapitulated for HLAs alleles of low frequency in West Africa, nor for the Mtb L4 strain variants (Table 3). Finally, no difference in the number of high-binding-affinity epitopes was predicted for the Rv2719c variant identified for the Mtb L4 strains as compared to the ancestral protein (Table 3). In summary, the epitope substitutions with high VAF in Mtb L6 were predicted to display an overall reduced binding affinity to both HLA class I and II alleles of high prevalence in West Africa. This pattern was not visible in the case of HLA alleles of low prevalence in West Africa, nor for Mtb L4 variants independently of the HLA allele frequency.

Thus, the findings so far support the hypothesis that Mtb L6 strains may have evolved to persist within a specific host genetic landscape, potentially explaining its confinement to West Africa. Of note, although we did not detect a difference in the number of high-binding-affinity peptides for RimJ when comparing Mtb L6 and the ancestral sequences, our in silico analysis predicted a loss of IFN-γ-producing peptides in the case of the Mtb L6 protein (Figure 3A). Similarly, a loss of IFN-γ-producing peptides was predicted for the Mtb L6 variant of LldD2 (Figure 3B). However, this loss of IFN-γ-producing peptides was not seen for Rv0012 and Rv0990c (Figure 3C,D), despite the decrease in the number of predicted high-binding-affinity peptides (Table 3). It is possible that different levels of adaptation exist to modulate T cell responses and confer a selective advantage by reducing immune detection while maintaining sufficient infectivity to ensure transmission within sympatric populations.

**Figure 4:**
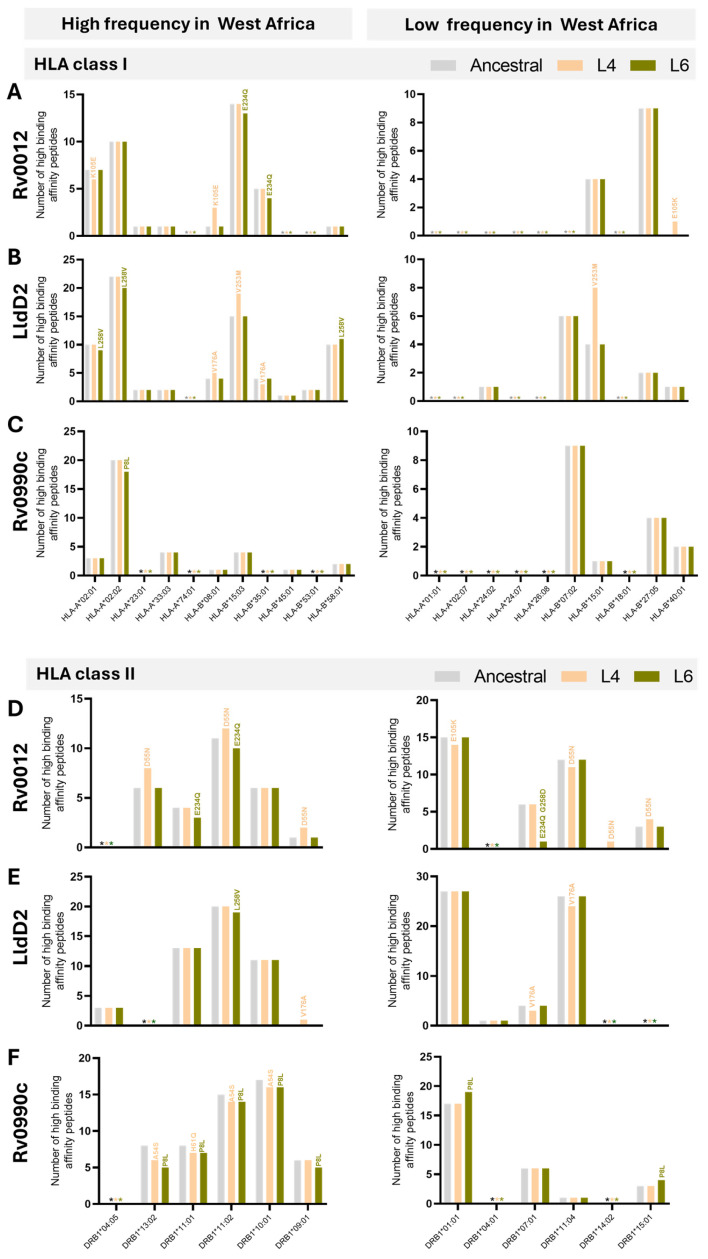
Predicted number of high-binding-affinity epitopes across lineages and HLA alleles. Number of high-binding-affinity epitopes predicted for Rv0012 (**A**,**D**), LldD2 (**B**,**E**), and Rv0990c (**C**,**F**) across the considered HLA class I (**A**–**C**) or HLA class II (**D**–**F**) alleles, as indicated on the x axis. Predictions for the ancestral sequences are represented in grey bars, for Mtb L4 variants in orange, and for Mtb L6 variants in green. Graphs on the left represent predictions for the indicated HLA alleles detected with high frequency in West Africa. Graphs on the right represent predictions for the HLA alleles detected with low frequency in West Africa. The non-synonymous substitutions that affect the number of high-binding-affinity predicted epitopes are indicated above each corresponding bar. Predictions were performed using NetMHCpan (HLA class I) and NetMHCIIpan (HLA class II), considering 8 to 14 mers for HLA class I and 9 to 18 mers for HLA class II. HLA frequencies were retrieved from allelefrequency.net. * (grey, orange, or green) indicates the absence of predicted high-binding-affinity peptides for the ancestral sequences, Mtb L4 variants, and Mtb L6 variants, respectively.

Given our analysis suggesting a possible match between variants of Rv0012, Rv0990c, and LldD2 in Mtb L6 strains and the populational HLA alleles, we next inspected with more detail the predictions obtained for these proteins with respect to the individual HLAs tested. In the case of Rv0012 Mtb L6 variants, the loss of two HLA class I high-binding-affinity peptides was caused by the mutation E234Q, specifically on HLA-B*15:03 and 35:01, both of high prevalence in West Africa (Figure 4A). This mutation in Rv0012 was also predicted to decrease the binding of peptides to HLA class II alleles HLA-DRB1*11:01 and HLA-DRB1*11:02, of high frequency in West Africa, and HLA-DRB1*07:01, of low frequency in West Africa (Figure 4D). In this protein, the Mtb L4 variants affected several HLA class I and II alleles, of high or low frequency in West Africa, with a global gain of predicted high-affinity peptides (Figure 4A,D). With respect to LldD2, the Mtb L6 variant L258V was predicted to display less high-binding-affinity epitopes to the high-frequency HLA class I alleles HLA-A*02:01 and HLA-A*02:02 and the class II HLA-DRB1*11:02 allele, whereas it associated with an increase in the number of high-binding-affinity peptides for the HLA-B*58:01 (class I) allele (Figure 4B,E). Mtb L6 variants of LldD2 did not impact the predicted binding to HLA class I or II alleles of low frequency in West Africa (Figure 4B,E), whereas Mtb L4 variants of this protein impacted the binding predictions to several HLA alleles, again with no specific pattern between high- or low-prevalence alleles and with a net gain in predicted high-binding-affinity epitopes (Figure 4B,E). Finally, the mutations affecting the Mtb L6 protein Rv0990c had only a minor effect on HLA class I (HLA-A*02:02) binding predictions (Figure 4C). However, both Mtb L6 and L4 Rv0990c variants widely impacted the predicted number of high-binding-affinity peptides in the context of HLA class II, specifically those of high prevalence in West Africa (Figure 4F). In both cases, a predicted loss in high-binding-affinity epitopes was detected (Figure 4F). Only the Mtb L6 variant of Rv0990c affected the binding to HLA class II alleles of low frequency in West Africa, in this case increasing the number of high-binding-affinity peptides (Figure 4F). Of note, the variants of Rv0012 (E234Q and G258D) and P8L of Rv0990c are represented in sub-lineage 6.2.3, whereas variants A68V of Rv0990c and L258V of LldD2 are broadly represented in L6 (Figure 2D). Therefore, it is tempting to speculate that the variant L258V of LldD2 might have the widest impact on the interaction of Mtb L6 strains with West African hosts. Future functional studies will need to take this potentially differential effect of antigen mutations in distinct sub-lineages into account.

Globally, whereas variations of Mtb L6 proteins in the predicted number of HLA class I high-affinity peptides did not affect a particular allele (within those selected for the study), in the case of HLA class II, the Mtb L6 mutations analyzed for the three selected antigens commonly affected the HLA-DRB1*11:02 allele, being invariably associated with a reduction in the number of high-affinity predicted epitopes. It would be interesting in the future to investigate whether individuals bearing this HLA class II allele show differences in susceptibility to TB disease or in the type and quality of CD4 T cell responses, particularly upon infection by Mtb L6 strains. Interestingly, in a study performed in East Asia, the HLA-DRB1*11 gene polymorphism was suggested to be a protective factor for TB risk [60]. Despite the many studies investigating possible association of HLA genetic variants with TB susceptibility [46,47,48,49,50,51,52,53,54], the results remain inconsistent. Our data highlight the genetic background of the infecting Mtb as a variable to consider in these studies. Supporting this view, a study conducted in 682 TB patients and 836 healthy controls in Thailand found an association of the HLA-DRB1∗09:01 and HLA-DQB1∗03:03 alleles with TB susceptibility in patients infected with modern Mtb strains and suggested strain specific susceptibility to TB [61]. Also relevant, only a few studies on HLA association with TB were performed in individuals of African ancestry, despite the high burden of TB in Africa [1].

Although outside of the scope of this study, experimental validation of the reported predictions is needed to confirm their functional impact. Future studies employing in vitro assays with peripheral blood mononuclear cells from West African individuals typed for specific HLA alleles will be crucial to elucidate the extent to which the variant Mtb L6 proteins may modulate immune responses in a natural infection setting. Moreover, expanding our analysis to additional Mtb antigens under positive selection in L6 could reveal broader immune evasion strategies that contribute to its epidemiological niche. Our study is based on the extensively validated pan-specific tools NetMHCpan and NetMHCIIpan. Also important as a valuable avenue for future work will be the development of a dedicated QSAR model.

## 4. Conclusions

The immunoinformatics-driven analysis presented in this study offers insights into the potential host–pathogen interactions in place between Mtb L6 strains and their preferred hosts. Focusing on a rare subset of highly variant Mtb antigens, we revealed possible alterations in binding patterns of Mtb L6 antigen variants to host HLAs that may underlie the restricted geographic distribution of Mtb L6. Further understanding the functional impact of these findings may have important implications for TB research, from vaccine design to genetic susceptibility studies, specifically those involving TB caused by Mtb L6 strains. It will be important in the future to experimentally validate the predictions presented in this study. That will include testing the immunogenicity of the Mtb L4 and L6 variant antigens in cells from TB patients stratified for the infecting bacteria (i.e., Mtb L4 or L6) and for their HLA allele. Also important will be the application of other in silico tools, such as QSAR, to improve the power of our predictions. Furthermore, integrating in silico predictions with high-throughput in vitro epitope screens or single-cell TCR repertoire analyses will expand our understanding of how antigenic variation shapes T cell recognition across populations. Coupling HLA–epitope binding data with genome-wide association studies (GWAS) holds the potential to uncover host genetic markers of susceptibility or resistance to Mtb L6, guiding lineage- or region-specific vaccine design and precision medicine strategies. Together, these approaches underscore the need to account for both pathogen diversity and host genetic background when developing next-generation TB control measures. Combined, this knowledge can also guide researchers in identifying broad-spectrum antigens that promote appropriate immune responses in different populations, increasing the effectiveness of universal vaccines. Collectively, this will contribute to improve TB control efforts in endemic regions.

## Figures and Tables

**Table 1:** HLA class I allele frequencies across five West African countries. Values represent the total number of copies of each allele in the population sample (Alleles/2n) in decimal format. Alleles are categorized into those with high or low prevalence in West Africa.

		Allele Frequency *				
		Guinea-Bissau	Mali	Senegal	The Gambia	Burkina Faso
HLAs with high prevalence in West Africa	HLA-A * 23:01	0.1858	0.228	0.177	n.d.	n.d.
	HLA-A * 02:01	0.1084	0.083	0.081	n.d.	0.0945
	HLA-A* 33:03	0.0882	0.094	0.081	n.d.	n.d.
	HLA-A * 74:01	0.13175	0.036	0.022	n.d.	0.032333
	HLA-A* 02:02	0.0714	0.076	0.091	n.d.	0.053
	HLA-B * 15:03	0.108	0.069	n.d.	n.d.	0.1045
	HLA-B * 35:01	0.144	0.127	0.122	n.d.	n.d.
	HLA-B * 53:01	0.1	0.159	0.053	n.d.	0.171
	HLA-B * 58:01	0.078	0.022	0.069	n.d.	n.d.
	HLA-B * 08:01	0.077	0.007	0.048	n.d.	n.d.
HLAs with low prevalence in West Africa	HLA-A * 24:02	0.0224	0	0.005	n.d.	n.d.
	HLA-A * 01:01	0.051	0.007	0.027	n.d.	n.d.
	HLA-A * 02:07	n.d.	n.d.	n.d.	n.d.	0.005
	HLA-A * 24:07	n.d.	n.d.	n.d.	n.d.	n.d.
	HLA-A * 26:08	0	0	n.d.	n.d.	n.d.
	HLA-B * 07:02	0.023	0.058	0.058	n.d.	n.d.
	HLA-B * 18:01	n.d.	0.0070	0.0270	n.d.	n.d.
	HLA-B * 27:05	0	0	n.d.	n.d.	n.d.
	HLA-B * 40:01	0	0	n.d.	n.d.	0.014
	HLA-B * 15:01	0	0	n.d.	n.d.	0

n.d. indicates that the allele was not detected in the respective population; * data retrieved from allelefrequency.net.

**Table 2:** HLA class II allele frequencies across five West African countries. Values represent the total number of copies of each allele in the population sample (alleles/2n) in decimal format. Alleles are categorized into those with high or low prevalence in West Africa.

		Allele Frequency *				
		Guinea-Bissau	Mali	Senegal	The Gambia	Burkina Faso
HLAs with high prevalence in West Africa	HLA-DRB1 * 11:01	0.1080	n.d.	0.0330	0.0846	n.d.
	HLA-DRB1 * 10:01	0.100	n.d	0.1890	0.0628	0.0900
	HLA-DRB1 * 04:05	0.0770	n.d.	n.d.	0.0475	n.d.
	HLA-DRB1 * 09:01	0.0770	n.d.	0.0330	0.0742	n.d.
	HLA-DRB1 * 11:02	0.0770	n.d.	0.1440	0.0753	n.d.
	HLA-DRB1 * 13:02	0.0770	n.d.	0.0440	0.1440	n.d.
	HLA-DRB1 * 15:03	0.0093	n.d.	0.0220	n.d.	n.d.
HLAs with low prevalence in West Africa	HLA-DRB1 * 04:01	0	n.d.	n.d.	0.0055	n.d.
	HLA-DRB1 * 07:01	n.d.	n.d.	0.0110	0.0557	n.d
	HLA-DRB1 * 11:04	n.d.	n.d.	n.d.	0.0005	n.d.
	HLA-DRB1 * 14:02	n.d.	n.d.	n.d.	n.d.	n.d.
	HLA-DRB1 * 15:01	0.0080	n.d.	n.d.	n.d.	n.d.
	HLA-DRB1 * 01:01	0	n.d.	0.0110	n.d.	n.d.

n.d. indicates that the allele was not detected in the respective population; * data retrieved from allelefrequency.net.

**Table 3:** Predicted differences in high-affinity HLA class I and II binding peptides for Mtb L6 and L4 amino acid variants vs. ancestral strain, using NetMHCpan and NetMHCIIpan, in the West African HLA frequency context.

**HLA Class I**			**Frequency in West Africa**			
			**High**		**Low**	
**Protein**	**L6 Variants**	**L4 Variants**	**L6–Anc.**	**L4–Anc.**	**L6–Anc.**	**L4–Anc.**
Rv0010c	A26V	-	0	-	0	-
RimJ	L123V	-	0	-	0	-
TB7.3	Q62H	-	−2	-	*	-
Rv0012	E234Q; G258D	D55N; K105E	−2	+1	0	+1
Rv0990c	P8L: A68V	A54S; H61Q	−2	0	0	0
LldD2	L258V	A59G; V176A; V253M	−2	+4	0	+4
Rv2719c	-	H124Y	-	0	-	0
**HLA Class II**			**Frequency in West Africa**			
			**High**		**Low**	
**Protein**	**L6 Variants**	**L4 Variants**	**L6–Anc.**	**L4–Anc.**	**L6–Anc.**	**L4–Anc.**
Rv0010c	A26V	-	−4	-	−5	-
RimJ	L123V	-	0	-	0	-
TB7.3	Q62H	-	0	-	0	-
Rv0012	E234Q; G258D	D55N; K105E	−2	+4	−5	0
Rv0990c	P8L: A68V	A54S; H61Q	−7	−5	+3	0
LldD2	L258V	A59G; V176A; V253M	−1	+1	+3	0
Rv2719c	-	H124Y	-	0	-	0

Orangecolor indicates loss of predicted high-binding-affinity peptides, yellow no difference, and green a gain for the Mtb L6 or L4 variant. *, indicates that no high-binding-affinity epitopes were detected for the HLA/protein pair.

## Data Availability

The data on variant allele frequencies are available in Supplementary Table S2. All genome sequences used for analyses were previously deposited online [39].

## Supplementary Materials

The following supporting information can be downloaded at: https://www.mdpi.com/article/10.3390/microorganisms13051032/s1, Table S1: List of oligonucleotides used in this study; Table S2: Mean VAF analysis across all lineages.

## Abbreviations

The following abbreviations are used in this manuscript:

CFU	Colony forming unit
HLA	Human leucocyte antigen
IC	Inhibitory concentration
IFN	Interferon
IL	Interleukin
L	Lineage
Mtb	*Mycobacterium tuberculosis*
MTBC	*Mycobacterium tuberculosis* complex
SNPs	Single nucleotide polymorphism
SVM	Support vector machine
TB	Tuberculosis
VAF	Variant allele frequency
