# Supplementary material for: Immunoinformatics Predictions on Variable Mycobacterium tuberculosis Lineage 6 T Cell Epitopes and HLA Interactions in West Africa
Source: Microorganisms. 2025 Apr 29;13(5):1032. doi: 10.3390/microorganisms13051032 (PMC12114075; doi:10.3390/microorganisms13051032)
Supplement: Supplementary file 1 [file microorganisms-13-01032-s001.zip › Silva_Supplemental Table S1.pdf]

Supplementary Table S1. List of oligonucleotides used in this study.

**Gene**	**Forward (5’-3’)**	**Reverse (5´-3’)**
Rv0012	GCCGCGTTGGGCTGACC	CCGTTGTGCGGATGACAC
Rv0010c	CGTCCGTTGACGATCAGC	GCGATGACGTTGTCGTTG
Rv0990c	GCGTTCGACGACATCGAC	CGACGTTGATGACGACGA
Rv1872c	GCGACGTTGACGATGACG	CGTGACGACGTTGACGAC
Rv3221c	GCGTTGACGACGATGACG	CGACGTTGACGACGTTGC
Rv2719c	CGCCGCTCGCTGAGCCGGC	CGGCCGCGGCGGATCGTC
Rv0995	GCGGCGATGCACCGCACG	CGCCGCCGCCGATGATCG
SigA	CAAGGACGCCGAACTCAC	CTT GCC GAT CTG TTT GAG
